# The evolution of floral deception in *Epipactis veratrifolia* (Orchidaceae): from indirect defense to pollination

**DOI:** 10.1186/1471-2229-14-63

**Published:** 2014-03-12

**Authors:** Xiao-Hua Jin, Zong-Xin Ren, Song-Zhi Xu, Hong Wang, De-Zhu Li, Zheng-Yu Li

**Affiliations:** 1 State Key Laboratory of Systematic and Evolutionary Botany, Institute of Botany, Chinese Academy of Sciences, Beijing 100093, China; 2 Key Laboratory for Plant Diversity and Biogeography of East Asia, Kunming Institute of Botany, Chinese Academy of Sciences, Kunming 650201, China; 3 University of the Chinese Academy of Sciences, Beijing 100039, China

**Keywords:** Anther cap, Aphids, Floral mimicry, Hoverflies, Intermediate stage, Pollinator

## Abstract

**Background:**

It is estimated that floral deception has evolved in at least 7500 species of angiosperms, of which two thirds are orchids. *Epipactis veratrifolia* (Orchidaceae) is a model system of aphid mimicry as aphidophagous hoverflies lay eggs on false brood sites on their flowers. To understand the evolutionary ecology of floral deception, we investigated the pollination biology of *E. veratrifolia* across 10 populations in the Eastern Himalayas. We reconstructed the phylogeny of *Epipactis* and mapped the known pollination systems of previously studied species onto the tree.

**Results:**

Some inflorescences of *E. veratrifolia* were so infested with aphids while they were still in bud that the some larvae of hoverflies developed to the third instar while flower buds opened. This indicated that adult female hoverflies were partly rewarded for oviposition. Although flowers failed to secrete nectar, they mimicked both alarm pheromones and aphid coloring of to attract female hoverflies as their exclusive pollinators. Phylogenetic mapping indicate that pollination by aphidophagous hoverflies is likely an ancestral condition in the genus *Epipactis*. We suggest that the biological interaction of aphid (prey), orchid (primary producer) and hoverfly (predator) may represent an intermediate stage between mutualism and deception in the evolution of pollination-by-deceit in *E. veratrifolia.*

**Conclusions:**

Our analyses indicate that this intermediate stage may be used as a model system to interpret the origin of oviposition (brood site) mimicry in *Epipactis*. We propose the hypothesis that some deceptive pollination systems evolved directly from earlier (partly) mutualistic systems that maintained the fidelity of the original pollinator(s) even though rewards (nectar/ brood site) were lost.

## Background

Most flowering plants depend primarily on animals for sexual reproduction, offering edible or non-edible rewards to their pollen vectors [1-3]. However, some “deceptive flowers” offer no rewards [4-6]. It is estimated that deceptive pollination systems occur in at least 7500 extant angiosperm species but at least two thirds of these species are in the family Orchidaceae [4-7].

Several hypotheses, including perceptual exploitation of pollinator cognitive/sensory bias and floral mimicry, have been proposed to understand the evolutionary pattern and mechanism of floral deception [5,8-11]. Recent studies indicate that pollinator perceptions and preferences for certain visual and olfactory cues are much older than some angiosperm lineages that currently offer these cues [10-12], and that pollination systems shifted numerous times between floral deception and rewards within a tribe or a genus [13-15]. Hobbhahn et al. [16] even suggested that the transition from no-reward to nectar rewards is not necessarily accompanied by visible morphological changes but only subcellular modifications in the genus *Disa*. Such observations have contributed much to our understanding on evolutionary patterns of floral deception; however, few efforts try to establish the evolutionary process of floral deception, and there is still little knowledge about these [17].

Brood-site mimicry is dependent on deceiving female insects seeking an oviposition site. This pollination system evolved independently in several unrelated angiosperm lineages including the Araceae, Aristolochiaceae, Asclepiadaceae and Orchidaceae [18]. Beetles and flies that typically oviposit on carrion, dung or the fruiting bodies of fungi are duped into laying eggs on a plant [19]. It is estimated that 11 genera of deceptive orchids, including *Epipactis* and *Paphiopedilum*, produce flowers with this mode of deceit [8,20].

Recent results indicate that *Epipactis veratrifolia* fools aphidophagous hoverflies by visual and olfactory floral signals [21,22]. Ivri & Dafni [21] suggested that the black callus-like swellings on the hypochile of the labellum mimic the aphids that are found infrequently on the vegetative organs of the same species. Stökl et al. [22] found that flowers of *E. veratrifolia* also mimicked an aphid alarm pheromone by producing α- and β-pinene*,* β*-*myrcene, and β*-*phellandrene.

Our preliminary field investigation in the Eastern Himalayas from 2009 to 2010 revealed that inflorescences of *E. veratrifolia* along the banks of the Salween River were often parasitized heavily by aphids both while in bud and during blooming. These aphids were similar in shape and color to the orchid’s anther caps. We also observed that hoverflies visited the flowers and removed the pollinaria. (See Supporting Information, Additional file 1: Table S1, Additional file 2: Figure S1A, and Additional file 3: Figure: S2 for details.)

Using *E. veratrifolia* as a model, this study attempts i) to define interactions among the orchids, aphids and predatory hoverflies; ii) to understand the evolution of floral deception in *E. veratrifolia*.

## Results

### Pollinators, eggs and larvae

The flowers of *E. veratrifolia* were visited primarily by females representing three species in the family, Syrphidae: *Eupeodes corollae*, *Episyrphus baleatus* and one unidentified species. During 114 observation hours, we recorded 129 visits by *Eupeodes corollae* (n = 112 visits), *Episyrphus baleatus* (n = 11) and the unidentified species (n = 6)*.* Floral visitation usually peaked between 15:00 and 17:00. Most syrphid species were observed carrying pollinaria on the dorsum of their thoraces. The most important pollinator appeared to be *Eupeodes corollae* based on its relative abundance and the high proportion of individuals carrying pollinaria.

**Figure 1:**
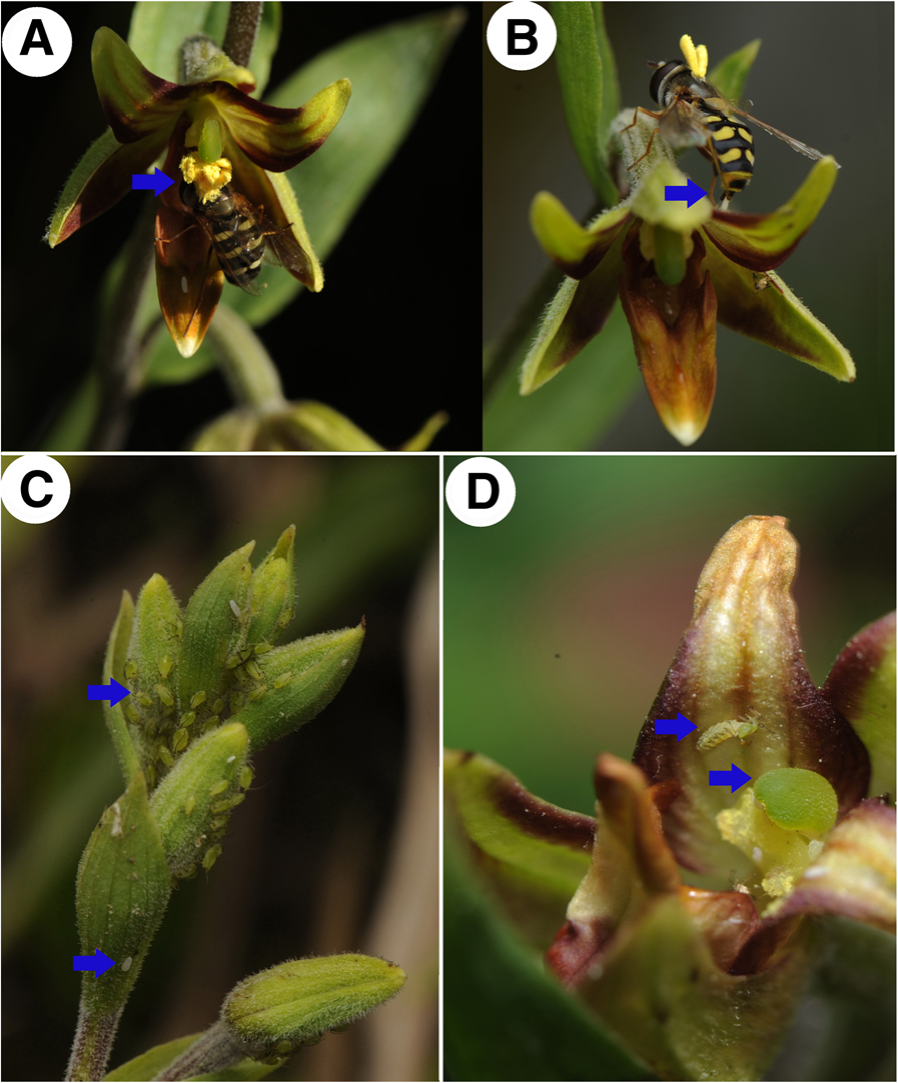
**Aphids hoverflies (adults and maggots) on buds and flowers. (A)** A hoverfly on a flower transporting the pollinaria on its dorsal thorax (arrow indicates position of pollinia). **(B)** Hoverfly carrying pollinia while laying an egg on a flower (arrow indicates the egg). **(C)** Aphids on inflorescences flowering in March (arrows indicate aphids and a hoverfly egg). **(D)** A second instar maggot preying on aphids on a flower (arrows indicate hoverfly instar and anther cap). Scale interpretation: **A** and **B**, average length of hoverfly = 9–10 mm; **C**, average length of aphids = 1 mm; **D**, average length of anther cap = 3 mm.

A total of 453 syrphid flights between flowers were recorded, including multiple visits to flowers on the same inflorescence by the same female. More than half of the recorded specimens of *Eupeodes corollae* carried 1–3 pollinaria (Figure 1A, B). Specifically, *E. corollae* flew to a flower, hovered, then landed on the epichile of the labellum (see epichile in Additional file 2: Figure S1B). We observed probing activity by some flies on the two transparent calli and the black calli on the hypochile of the labellum (see hypochile in Additional file 2: Figure S1B). Visitation behavior by *E. baleatus* was similar. The transfer of pollinia fragments to the receptive stigma occurred when a pollinarium-bearing insect crawled toward the hypochile, located under the column, and then backed out. Backing out also transferred a fresh pollinarium to the pollinator’s dorsum.

**Figure 2:**
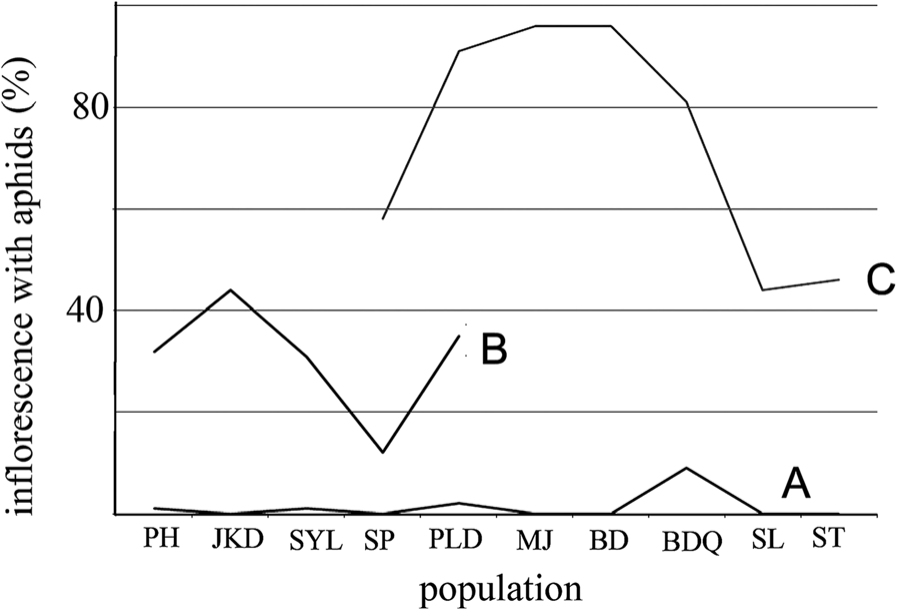
**Percentage of inflorescences with hoverfly eggs. (A)** The percentage of inflorescences in bud in March bearing eggs. **(B)** The percentage of inflorescence with open flowers in March bearing eggs. **(C)** The percentage of inflorescences with open flowers in April bearing eggs.

The number of hoverfly eggs on each flower ranged from 0–13. During the first field survey (March 7–17, 2012), five out of the 669 sampled budding inflorescences had 13 eggs in ten populations (0.74%). In contrast, 154 out of the 340 blooming inflorescences (45.3%) had a total of 314 hoverfly eggs. During the second survey (April 11–13, 2012), hoverfly eggs were found on 413 out of the 632 sampled blooming inflorescences (65.3%), with a total of 1190 eggs. Egg deposition rates differed significantly among the three types of inflorescences sampled (ANOVA, F_2, 1839_ = 35.768, P < 0.001; Figure 2). The number of eggs per inflorescence was not significantly different between flowering inflorescences in March (mean 2.03 eggs/inflorescence) and April (2.85 eggs/inflorescence; ANOVA, F_1, 1042_ = 2.748, P = 0.126).

We observed three instar stages of hoverfly maggots in all three orchid populations (Figure 1D, Additional file 2: Figure S1C) but third instar maggots were rare (only seven observed). We also observed maggots preying on aphids (Figure 1D). By the third instar, maggots crawled freely among inflorescences, presumably to search for prey. Maggots pupated following the third instar but these pupae dropped to the ground and we were unable to recover them.

### Aphid observation

Inflorescences, flower buds and flowers of *Epipactis veratrifolia* were infested with aphids. Morphological characteristics and DNA barcoding identified the aphids as *Aulacorthum solani.* This species was present on inflorescences in all 10 orchid populations*.* Wingless females gave birth to live young (Additional file 2: Figure S1C, D) and colonies were concentrated on flower buds and scapes (Figure 1C; Additional file 1: Tables S2–4). Individual aphids were also found on open flowers (Additional file 2: Figure S1C & D), but not on plants in a vegetative state.

The frequency of aphid infestation was not significantly different among the three types of inflorescences observed across 10 populations (ANOVA, F_2, 22_ = 0.219, P = 0.805), including budding inflorescences (10.8%), March-flowering inflorescences (9.7%), and April-flowering inflorescences (8.9%). There was also no significant difference in the occurrence of aphids on flowers open in March compared with flower buds (F_1, 212_ = 0.167, P = 0.683). In contrast, the number of aphids per parasitized inflorescence was significantly different among the three types of inflorescences (F_2, 18_ = 6.058, P = 0.011). Budding inflorescences had an average of 8.85 aphids, whereas there were 19.86 in March-flowering inflorescences, and 2.24 in April-flowering inflorescences.

### Breeding system of orchids

The bagged control flowers failed to produce fruit. Fruit set in hand-pollinated self- and cross-pollination flowers was 93.2% and 100% (Table 1). The fruit set of open, insect-pollinated flowers was 45.3% ± 0.232 (mean ± SD, 4709 flowers in 741 inflorescences) in 2012 and differed significantly among the nine orchid populations (F_8, 733_ = 5.449, P < 0.001) (Table 2).

**Table 1:** **Breeding system of *Epipactis veratrifolia *** **in 2011**

**Treatment**	**No. inflorescences**	**No. flowers**	**No. capsules**	**Fruit set (%)**
Bagged (control)	11	40	0	0
Cross-pollination	10	39	39	100
Self-pollination	10	30	28	93.33

**Table 2:** **Natural fruit produced by *Epipactis veratrifolia *** **in 2012**

**Population**	**No. inflorescences**	**No. flowers**	**No. capsules**	**Fruit set (%)**	**SD**	**SE**
BD	102	651	262	40.2	0.22356	0.02214
BDQ	33	246	85	34.5	0.19979	0.03532
JKD	102	325	109	33.5	0.24671	0.02443
MJ	102	722	263	36.4	0.20352	0.02015
PH	100	733	402	54.8	0.23865	0.02620
PLD	102	662	297	44.9	0.22773	0.02255
SL	102	587	274	46.7	0.23568	0.02334
SP	102	713	355	49.8	0.21395	0.02118
ST	25	44	21	47.7	0.25573	0.05115

### Volatile composition of flowers vs. aphids and floral nectar

**Figure 3:**
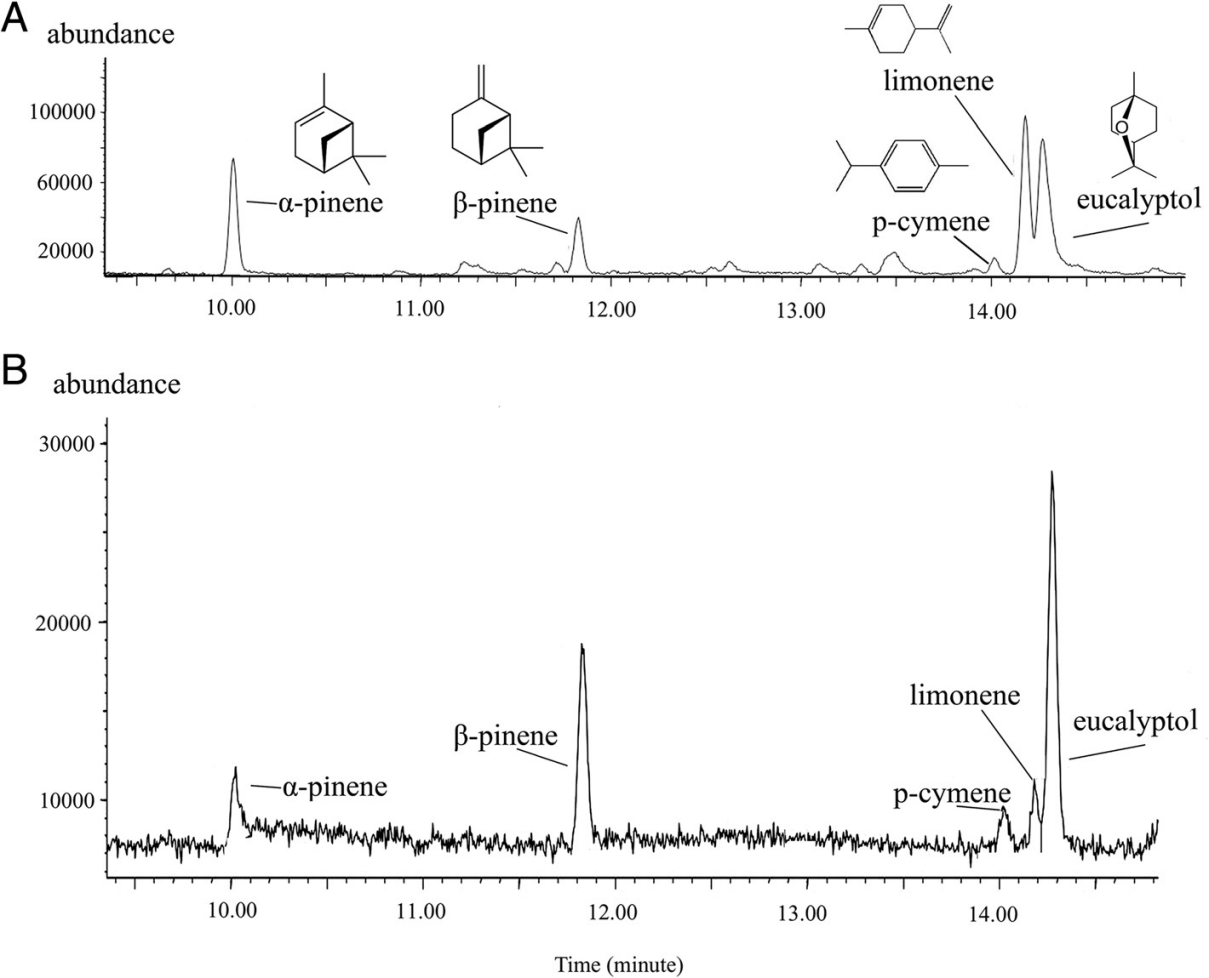
**GC-MS traces of (A) headspace volatiles of the flower of *Epipactis veratrifolia*; (B) surface extract of specimens of *Aulacorthum solani.***

Five volatiles were detected using GC-MS in headspace collections of flowers of *E.veratrifolia*: α-pinene (comprising 20.76% of the total samples), β-pinene (10.61%), limonene (29.86%), eucalyptol (38.75%), and trace amounts of p-cymene (Figure 3A). The surface extracts from aphids (*A. solani*) contained α-pinene (9.23%), β-pinene (25.93%), p-cymene (8.9%), limonene (8.30%), and eucalyptol (47.47%) (Figure 3B).

No nectar was found in any of the inflorescences sampled, nor could we detect nectar droplets using light microscopy.

### Bioassay experiments and reflectance

The synthetic odor mixture triggered eight approaches by hoverflies to budding inflorescences. Three of these flies contacted buds. In contrast, only one fly approached the control inflorescences and it did not make contact (For approach, χ^2^ = 9.89, df = 1, P = 0.003).

**Figure 4:**
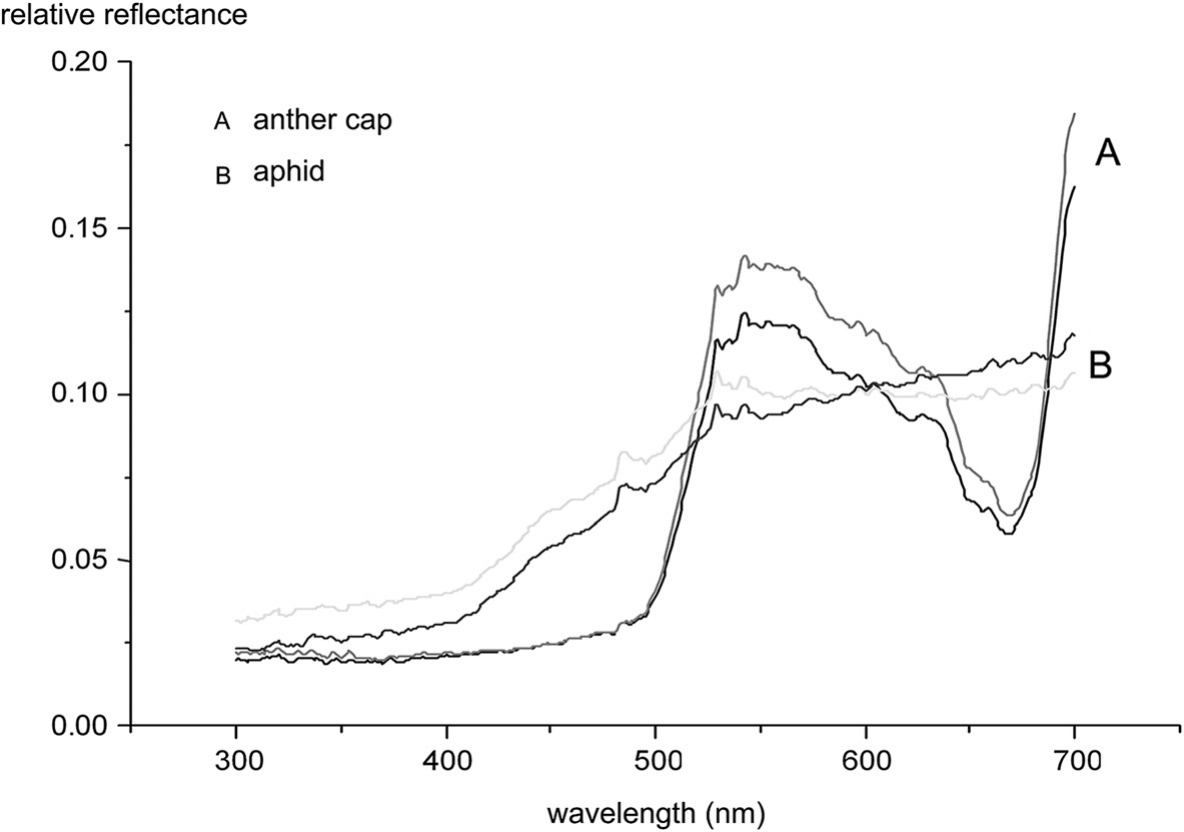
**The reflectance of aphids and anther caps. (A)** Reflectance of anther caps, two lines representing two replicates. **(B)** Reflectance of aphids, two lines representing two replicates, each on a group of aphids.

The spectral peak of both the anther cap and the aphid bodies began at 500 nm (Figure 4).

### Phylogenetic structure and evolution of pollination systems of *Epipactis*

**Figure 5:**
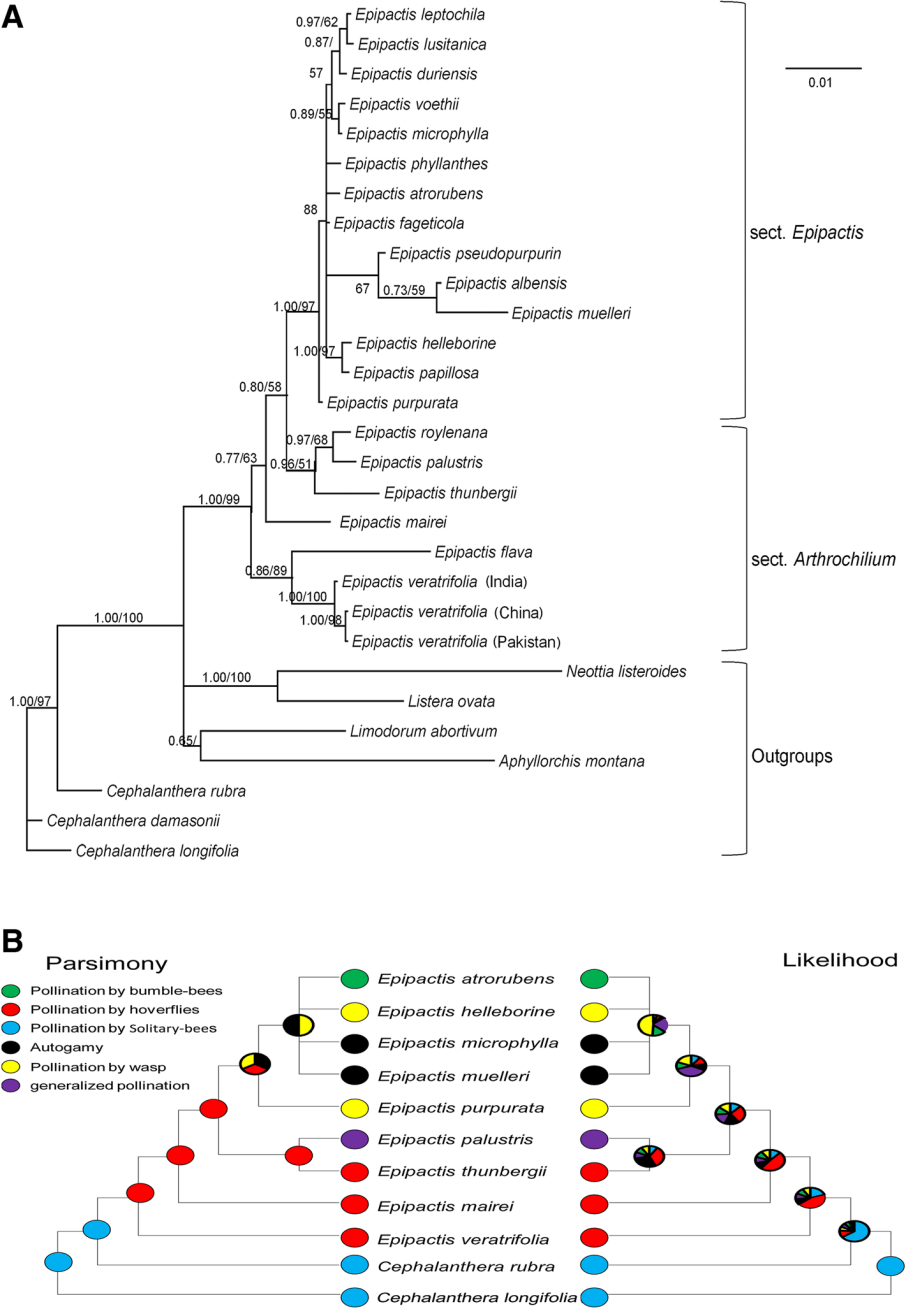
**Phylogeny and evolutionary pattern of pollination system of *Epipactis. *(A)** Phylogram of the *Epipactis* lineage. **(B)** Mapping of pollination systems in *Epipactis* onto the phylogram. Numbers at the nodes are Bayesian posterior probabilities and bootstrap percentages (>50%).

Phylogenetic analyses of the combined, three-region DNA sequence generated a highly resolved and well-supported lineage. The genus *Epipactis* was monophyletic with strong support (Posterior Probabilities (PP) = 1.00, Bootstrap (BS) = 100) with *Cephalanthera* as the immediate outgroup. Section *Arthrochilium* was paraphyletic with sect *Epipactis* deeply nested within it (Figure 5). Section *Epipactis* was monophyletic with strong support (PP = 1.00, BS = 95) with 13 species forming a polytomy sister group to *E. purpurata. Epipactis veratrifolia* and *E. flava* (sect. *Arthrochilium)* formed their own clade as a sister group to the remaining 18 species of *Epipactis*.

According to previous pollination studies on *Epipactis* and the phylogenetic relationships (Figure 5), the ancestral reconstruction suggested that pollination by (female and/or male) hoverflies is likely ancestral in *Epipactis*. The aphidophagous-hoverfly pollination system is restricted currently to sect. *Arthrochilium* (*E. veratrifolia* and *E. thunbergii*) based on incomplete sampling of the genus.

Although the pollination system of *Epipactis flava* is unknown, it is the sister species of *E. veratrifolia* and shares the same floral presentation [23]. Most of the self-pollinating (autogamous) species in sect. *Epipactis* were not included in this molecular analysis; however, sect. *Epipactis* was deeply embedded in sect. *Arthrochilium* and derived in *Epipactis*. We did not have samples of the hoverfly pollinated, *E. gigantea*, however, it is considered as identical in floral traits to as *E. royleana*[24]. We believe these three factors are likely to have little effect on our results.

## Discussion

### Biological interactions between pollinators and orchids

Biological interactions among orchids, their pollinators, and their parasites in the Eastern Himalayas (EH) were subdivided into two different life stages: budding stage vs. the open flower stage. During the budding stage, large numbers of aphids infested some inflorescences (Figure 1C, Additional file 1: Table S2) but few hoverfly maggots were present on their buds. In contrast, during the open flower stage, after the majority of aphids were consumed by the earlier- hatched hoverfly maggots, the inflorescences were now covered in fresh hoverfly eggs laid by much later- arriving females.

Although the Eastern Himalayas (EH) are located far from the Mediterranean basin (MB), *E. veratrifolia* is pollinated by aphidophagous hoverflies in both regions. However, we observed striking variation among these populations, including type of floral rewards, natural fruit set, pollinator biological interactions and the components of floral volatiles (Table 3). Specifically, flowers offer a small amount of nectar as rewards to pollinators in MB whereas in the EH, flowers offer live aphids as rewards to some pollinators. Our results indicate that populations of *E. veratrifolia* in the EH could only offer a declining number of aphids to the larvae of its pollinators because the majority of these larvae hatched after the flowers opened. It seems likely that most larvae hatching on blooming inflorescences must have starved to death as first instar maggots could not crawl very far to find aphids. Therefore, the interaction between the orchid and the hoverfly in EH is partly mutualistic.

**Table 3:** **Intraspecific variation in the pollination characteristics of *E. veratrifolia*** **in the Eastern Himalayas and Israel (Ivri & Dafni, 1977). + = character present; - = character absent**

**Characteristic**	**Israel**	**Eastern Himalayas**
Anther cap green	+	+
Aphids black	+	-
Aphids green	-	+
Nectar secretion	+	-
Natural fruit set	0.155	0.33 –0.548

### Floral mimicry

Insects discriminated colors based on wavelength differences of spectral peaks [19,25], and an increasing number of studies indicate that visual signal may dominate a pollinator’s choice of a flower at short distances [20,26,27]. Our results suggest that *E. veratrifolia* flowers may mimic two common aphid colors, green and black (for aphids color, see http://www.aphidsonworldsplants.info/), throughout its range. In Israel, for example, hypochile of flowers of *E. veratrifolia* are ornamented with black callus-like swellings and are believed to mimic native black aphids [21]. Our results on reflectance and the anther cap removal experiment indicated that the green anther cap might also mimic green aphids as caps have a similar appearance to *A. solani* in both color and shape (Figures 1C, D; Figure 4). However, further studies should confirm this.

### Pollination system evolution in *Epipactis*

The ancestral reconstruction suggested that species pollinated by hoverflies are the basal group in *Epipactis*, and *E. veratrifolia* and *E. flava* are sister groups to the remaining of *Epipactis*. This suggests that the pollination system incorporating live aphids, predatory maggots and winged hoverflies may be ancestral within *Epipactis* (Figure 5B). Thus far, this is the only *Epipactis* species shown to offer live aphids to its pollinators in bud and in flower as aphids are not directly involved in tritrophic biological interactions in MB (Table 3). Based on these results, we suggest that this tritrophic interaction (pollinator/larval predator-orchid-aphid) may represent an intermediate stage between plant-predator mutualism, or indirect defense (see [28-30]),) and floral deception.

## Conclusion

Our results indicate that biological interactions between the orchid (*Epipactis veratrifolia*) and their syrphid pollinators (hoverflies) in the Eastern Himalayas are partly mutualistic. The tritrophic interaction (pollinator/larval predator-orchid-aphid) may be ancestral within *Epipactis* and may be an intermediate stage between plant-predator mutualism (or indirect defense) and floral deception. We propose a hypothesis that a fully deceptive mode of pollination may evolve directly from mutualistic or partially mutualistic systems that maintained the fidelity of the original pollinator(s) even though rewards were lost.

## Methods

### Study species

*Epipactis veratrifolia* (syn. *E. consimilis*) is a medium-sized, terrestrial geophytic orchid with a wide distribution, from northern Africa through the southern Mediterranean basin eastwards to the Himalayas. It occupies a diverse range of habitats, from humid limestone soils to the flood zones along riverbanks and elevations of 200–3000 m [23,24,31]. It flowers from early March to early May. Each inflorescence produces 1–18 flowers (mean ± SD, 6.9 ± 3.4, n = 53)*.* Flowers bloom acropetally over several days with two or three flowers opening simultaneously along the scape.

### Study sites

We used 10 accessible, randomly distributed populations of *E. veratrifolia* located in the flood zones along the banks of the Salween River (Additional file 1: Table S1, Additional file 2: Figure S1A and Additional file 3: Figure S2) from 2009 to 2013. All populations were subject to river overflow during monsoon season (July–September).

### Observations of pollinators, eggs and larvae

Flower visitors were observed between 7:00 and 19:00 from 22 April–5 May 2011 in ST and SL sites (137 flowers on 20 flowering stems), and from 5 March– to20 May 2012 in BD, PLD, SYL, JKD and PH sites (452 flowers on 50 flowering stems). Observation time totaled 114 hours. We recorded each visitor’s species, behavior (including egg laying), the time it visited each flower and inflorescence, and the number of pollinaria attached to it.

First-stage and third-stage instars of hoverflies were investigated in the SL site in April 2011 and in PH and JKD sites in March 2012 to determine whether hoverfly larvae were able to metamorphose into pupae. From 23 March to 8 April 2012, 100 inflorescences in bud were tagged at random and we recorded the presence/absence of aphids every two days on buds and open flowers*.*

### Observations of aphids

The occurrence of aphids was investigated to determine which species infested the orchids and where they were present (vegetative parts or inflorescences). We recorded the frequency of aphids and hoverfly eggs twice in all 10 populations in 2012, (first survey in March 7–17, second in April 11–13). Plants from each population were subdivided into three groups: (1) plants without inflorescences, (2) plants with inflorescences in bud and (3) plants with inflorescences with one or more open flowers. We surveyed all individuals in the 10 populations, except when population size exceeded 100 plants for each group. In these larger populations we selected 100 plants for each group at random. When we sampled the PH and JKD populations in April, which coincides with the end of the flowering season, the majority of inflorescences had ceased flowering.

### Identification of aphids and pollinators

Insects were collected from inflorescences of *E. veratrifolia*, and preserved in jars with ether fumes. Insects were identified using morphological characteristics, confirmed with DNA barcoding using cytochrome C oxidase I. Voucher specimens were deposited in the National Zoological Museum of the Institute of Zoology, Chinese Academy of Sciences (CAS).

### Nectar collection

Floral nectar in *E. veratrifolia* was examined in the SL population in 2011 and in the JKD population in 2012. We bagged 10 inflorescences at random in each population with nylon net bags before the buds opened. Following opening of each perianth, a 1–5-μl calibrated microcapillary tube (Sigma-Aldrich, St. Louis, MO, USA) was inserted by depressing the labellum and pushing it down the perianth tube to draw off nectar and record volume. Four additional flowers were removed to check the hypochile for the presence of nectar droplets under a dissecting microscope (Nikon, Japan).

### Volatile collections and analysis

Floral volatiles were collected in the field at 14:00 using dynamic headspace adsorption methods in the SL population in 2011. Volatiles of the aphids were collected from aphids found on the inflorescences. We placed 20 wingless aphids (1–2 mm in length but representing mixed-growth instars) in a clean glass vial containing 1 ml of pentane for 120 seconds. The volatiles were analyzed on a Hewlett-Packard 6890 Series GC System coupled to a Hewlett-Packard 5973 Mass Selective Detector using an Agilent 7683 Series Automatic Liquid Sampler [32]. (See Supporting Information for details of collection and analyses)

### Bioassay experiments

A manipulative anther cap removal experiment was conducted in the SL population in April, 2011. Forty flowers on nine plants in one patch were bagged before buds opened. After the perianth opened, we gently removed each anther cap with forceps. The bag was removed permanently and exposed for natural pollination. An additional 36 flowers on nine plants in the same patch were observed as controls during the same period.

Behavioral experiments were performed in late March 2013 in the PLD population. All bioassays were conducted between 15:00 and 16:00, during peak of pollinator activity. We selected 10 inflorescences in bud that lacked aphids and belonged to plants at least 2 m from plants with open flowers. The buds on these plants were treated with a synthetic mixture of compounds identified previously from the headspace samples (51 ng μl^−1^ (±) α-pinene and 43 ng μl^−1^ (±) β-pinene (Sigma-Aldrich, St. Louis, MO, USA) [22]. For each experiment, a 4-cm-high brown bottle containing 2 ml of the synthetic mixture was placed at the base of each plant in bud. Bottles with an equal volume of pentane were used as a control. The behavioral response of pollinators to each treatment was observed for 20 min, and behavior was recorded as (1) approached at close range (hovered less than 5 cm from buds but did not touch them) or (2) touched buds.

### Spectral reflectance analysis of anther cap and aphids

Spectral reflectance (%) across the 300–700-nm range was measured during April 2013 using a USB2000 spectrometer (Ocean Optics) and a UV-vis fiber optic reflection probe (PX2) held at 90° and 5 mm from an aphid or anther cap surface. Aphids were removed from the inflorescences in the PLD population, and the anther caps were also removed from flowers in the same population. Two replicates were conducted for aphids and anther caps, respectively.

### Breeding systems

The breeding system was evaluated using controlled bagging experiments following Dafni et al. [33]. Treatments included manual self- and cross-pollination, and bagged, flowers that were not hand-pollinated (control, natural self-pollination). Each treatment included about 40 flowers on 10 plants. The pollinaria used for cross-pollination were collected from plants from patches at least 10 m apart. Natural fruit set was recorded in nine populations in 2012 but the SYL population was destroyed due to a road construction project in 2012 before fruit set could be recorded.

### Molecular phylogenetics and the evolution of *Epipactis* pollination systems

The genus *Epipactis* Zinn. (Orchidoideae) is distributed primarily through temperate Eurasia with a few species endemic to tropical Africa and North America [34]. Descriptions of insect-pollination in *Epipactis* began in the 19^th^ century and continue to the present day [22,35,36]. The genus consists of 15–65 species, and is subdivided into two sections; sect. *Arthrochilium* and sect. *Epipactis*[37]. Sect. *Epipactis* consist of 10–60 species while and sect. *Arthrochilium* (including *E. veratrifolia*) contains seven to eight species. *Epipactis* was considered as taxonomic problem because of delimiting autogamous and agamospermous populations in sect. *Epipactis*[38,39]. To represent both sections, a total of 20 Eurasian species, six from sect. *Arthrochilium* and 14 from sect. *Epipactis* (see Supporting Information, Additional file 1: Table S5), were sampled. Three samples of *E. veratrifolia* were included. We sequenced chloroplast *rbcL*, *matK*, and nuclear ITS markers, and analyzed them with Most Parsimony and Bayesian Inference. The pollination systems of these species, based on the literature located on published books, Google Scholar and Web of Science, were referenced to map onto the tree following a maximum parsimony approach using Mesquite v2.74 [21,22,36,40-46]. Details of the molecular phylogenetics and the reconstruction of the *Epipactis* lineage are presented in the Supporting Information.

### Statistical analyses

Statistical analyses were performed in SPSS 16.0 for Windows. Natural fruit set, aphid infestation and hoverfly egg deposition were analyzed with one-way analysis of variance (ANOVA). A chi-squared test was used in bioassay experiments.

## Abbreviations

BD: Ben-dan population/site; BDQ: Ben-dan-qiao; BS: Bootstrap; EH: Eastern Himalayas; JKD: Jia-ke-ding; MB: Mediterranean basin; MJ: Ma-ji; PH: Pi-he; PLD: Pu-la-ding; PP: Posterior Probabilities; SL: Shuan-la; SP: Shan-pa; ST: Song-ta; SYL: Shi-yue-liang.

## Competing interests

The authors declare that they have no competing interests.

## Authors’ contributions

XHJ, ZXR, HW and DZL conceived and designed the experiments. XHJ, SZX and ZXR performed the experiments. XHJ and ZXR analyzed the data. XHJ and ZXR wrote the manuscript. HW, DZL and ZYL revised the draft. All authors read and approved the final manuscript.

## Supplementary Material

Additional file 1 **Table S1.** Locality of each population (elevation, m; population size, plants/inflorescences, data collected in May, 2012). **Table S2.** Aphids on plants with budding inflorescence during the first survey (March 7-17, 2012). **Table S3.** Aphids on plants with blooming inflorescence during the first survey (April 11–13, 2012). **Table S4.** Aphids on plants with blooming inflorescence during the second survey. **Table S5.** Taxa, voucher and GenBank accession numbers of Epipactis used in this study. **Table S6.** Primers used for amplification in this study. **Table S7.** Pollination systems of *Epipactis* and *Cephalanthera*. **Table S8.** Statistics from the analyses of the various datasets. Click here for file

Additional file 2: Figure S1 Habitat and floral organs of *Epipactis veratrifolia*. **A**) Habitat of *E. veratrifolia* along the Salween bank; **B**) hypochile, epichile, column and anther cap of *E. veratrifolia,* arrow indicating anther cap; **C**) Larva on dorsal sepal, aphid on lateral sepal (arrows indicate aphid and larva); **D**) Aphids and egg on flowers (arrows indicate aphids and egg. For sense of scale, **A**, the plant in bloom averages 40-60 cm in height; **B**, the length of anther cap averages 3 mm; **C**, the dorsal sepal averages 12 mm; **D**, egg length averages 0.7 mm. Click here for file

Additional file 3: Figure S2 Distribution of *E. veratrifolia* in Eastern Himalayas along Salween. Click here for file
