# Supplementary material for: The evolution of floral deception in Epipactis veratrifolia (Orchidaceae): from indirect defense to pollination
Source: BMC Plant Biol. 2014 Mar 12;14:63. doi: 10.1186/1471-2229-14-63 (PMC4007573; doi:10.1186/1471-2229-14-63)
Supplement: Additional file 1 — Table S1. Locality of each population (elevation, m; population size, plants/inflorescences, data collected in May, 2012). Table S2. Aphids on plants with budding inflorescence during the first survey (March 7-17, 2012). Table S3. Aphids on plants with blooming inflorescence during the first survey (April 11–13, 2012). Table S4. Aphids on plants with blooming inflorescence during the second survey. Table S5. Taxa, voucher and GenBank accession numbers of Epipactis used in this study. Table S6. Primers used for amplification in this study. Table S7. Pollination systems of Epipactis and Cephalanthera. Table S8. Statistics from the analyses of the various datasets. [file 1471-2229-14-63-S1.docx]

**Supporting Information**

Evolution of floral deception in *Epipactis veratrifolia* (Orchidaceae): from indirect defence to pollination

Xiao-Hua Jin^1*^, Zong-Xin Ren^2^, Song-Zhi Xu^1,3^, Hong Wang^2^, De-Zhu Li^2^, Zheng-Yu Li^1^

^1^State Key Laboratory of Systematic and Evolutionary Botany, Institute of Botany, Chinese Academy of Sciences, Beijing 100093, China.

^2^Key Laboratory of Biodiversity and Biogeography, Kunming Institute of Botany, Chinese Academy of Sciences, Kunming 650201, China

^3^ University of the Chinese Academy of Sciences, Beijing 100039, China

*Corresponding authors, emails: [xiaohuajin@ibcas.ac.cn](mailto:xiaohuajin@ibcas.ac.cn%20)

**Molecular Phylogenetics and the evolution of pollination systems of *Epipactis***

A total of 20 species (Table S5), representing two sections; sect. *Epipactis* and sect. *Arthrochilium*, were included. Three samples of *Epipactis veratrifolia* were used. Outgroups included five species from tribe Neottieae (Orchidoideae; Orchidaceae). We sequenced chloroplast *rbcL*, *matK* and nuclear ITS markers. Voucher information and GenBank accession numbers are listed in Table S5. Total genomic DNA was isolated from silica-gel-dried materials using a Plant Genomic DNA Kit (Beijing Biomed Co., LTD, Beijing, China). The PCR primers for matK, rbcL, and ITS are listed in Table S6. The selected DNA regions were amplified by using a standard polymerase chain reaction (PCR). The sequencing reactions were performed by using the ABI Prism Bigdye Terminator Cycle Sequencing Kit (Applied Biosystems, ABI).

Sequences were aligned using the default parameters in Clustal X version 1.83 [[1](#_ENREF_1)] and manually adjusted with BioEdit version 5.0.9 [[2](#_ENREF_2)]. Phylogenetic analyses for the combined dataset were carried out using parsimony (PAUP version 4.0b10) [[3](#_ENREF_3)], and Bayesian inference (BI; MrBayes version 3.1.2) [[4](#_ENREF_4)]. The homogeneity between the nrDNA ITS data and the combined plastid dataset (*rbcL* and *matK*) was tested using the incongruence length difference (ILD) test (Farris et al., 1995), implemented in PAUP version 4.0b10[[3](#_ENREF_3)]. For MP, parsimony heuristic searches were performed with 1000 random sequence addition replicates, tree-bisection-reconnection (TBR) branch swapping, MulTrees in effect. Steepest descent was not used. Internal branch support under MP was estimated by using 1000 bootstrap (BS) replicates. For BI, the analysis was performed with two runs, each having four chains, with trees and parameters sampled every 1000th generation. Burn-in and convergence were assessed using the likelihood of the runs plotted against generations using Tracer version 1.5 (http://beast.bio.ed.ac.uk/Tracer). Trees were summarized after removing the burn-in samples. Finally, a 50% majority-rule consensus tree was built in MrBayes.

Pollination systems of *Epipactis* were investigated using published books, Google Scholar and Web of Sciences [[5-14](#_ENREF_5)] . In total, pollination systems of nine species have been investigated previously and were included in the analysis (Table S7). The ancestral systems of *Epipactis* were reconstructed under a maximum parsimony approach in Mesquite version 2.74. (http://mesquiteproject.org/mesquite/mesquite.html).

This data set included nrITS and plastid (*rbcL* and *matK*) for 29 taxa. The ITS dataset consisted of 631 characters of which 86 (19.2%) were parsimony informative, and the chloroplast dataset consisted of 2919 characters of which 35 (0.1%) were parsimony informative (Table S8). According to the partition homogeneity test, the two datasets were congruent (*P* = 0.11). Therefore, we combined the two datasets for the phylogenetic analysis. Bayesian trees of each dataset were congruent with the MP strict tree, except for the weakly supported nodes.

*Epipactis* was monophyletic with strong support (Posterior Probabilities = 1.00, Bootstrap = 100). Despite the morphological similarities within sect. *Arthrochilum* this section was paraphyletic. *Epipactis veratrifolia* and *E. flava* of sect. *Arthrochilium* formed a well resolved clade that was the sister to the remaining *Epipactis* species. Sect. *Epipactis* deeply nested within sect. *Arthrochilum* and was supported as a monophyletic clade (PP = 1.00, BS = 95) (Figure 5).

**Volatile collections and analysis**

Floral scent was collected in the field at 14:00 using dynamic headspace adsorption methods in SL in 2011. One inflorescence with newly opened flowers that was free from aphids was enclosed in a polyethylene terepthalate bag (25 × 38 cm; Sainsbury’s Supermarkets). Two holes were cut at opposite ends of the bag. One hole was fitted with an activated carbon filter (Supelco) for air intake, and the other was fitted with a Super-Q volatile collection trap (Analytical Research Systems) containing 30 mg of Alltech Super-Q adsorbent material. Each flower was enclosed for 2h, after which the flower headspace was sampled with two micropumps driven by a portable battery. Constant airflow was adjusted to 100 mL/min using a flow meter. The sampling periods were 3–4 h. Empty cooking bags placed in close proximity to the flower were sampled as controls. After fragrance sampling, adsorbed volatiles were eluted from the Super-Q with 0.15 mL of dichloromethane (Uvasol; Merck). Samples were sealed in glass vials and stored at −20 °C.

The volatiles were analyzed on a Hewlett-Packard 6890 Series GC System coupled to a Hewlett-Packard 5973 Mass Selective Detector using an Agilent 7683 Series Automatic Liquid Sampler. An HP-5MS column (5% phenylmethylpolysiloxane; 60 m long, 0.32 mm inner diameter, 0.25 μm film thickness; Agilent) was used for the analyses. Electronic flow control was used to maintain a constant helium gas flow of 1.4 mL/min. The GC oven temperature began at 50 °C and was increased by 5 °C/min to 100 °C and held for 10 min, then increased by 5 °C/min to 280 °C and held for 5 min. The MS interface was 280 °C, and the ion trap was activated at 150 °C. The mass spectra were taken at 70 eV (in EI mode) with a scanning speed of 1 per scan from m/z 35–550. Component identification was performed using the Wiley NIST 05 mass spectral database and Wiley 7.

**Table S1 Locality of each population （elevation, m; population size, plants/inflorescences, data collected in May, 2012）**

| Population | Latitude | Longitude | Elevation (m) | size |
| --- | --- | --- | --- | --- |
| Song-ta (ST) | 28°09′56.76″N | 098°29′00.88″E | 1650 | 500 plants, 300 inflorescences |
| Shuan-la(SL) | 27°59′02.00″N | 098°39′10.84″E | 1539 | 400 plants, 150 inflorescences |
| Ben-dan-qiao (BDQ) | 27°53′40.67″N | 098°40′31.70″E | 1479 | 100 plants,40 inflorescences |
| Ben-dan (BD) | 27°48′05.89″N | 098°41′02.03″E | 1457 | 1000 plants, 300 inflorescences |
| Pu-la-ding (PLD) | 27°34′31.61″N | 098°48′16.78″E | 1396 | 2000 plants, 800 inflorescences |
| Ma-ji (MJ) | 27°31′06.17″N | 098°49′43.09″E | 1364 | 400 plants, 200 inflorescences |
| Shi-yue-liang (SYL) | 27°20′36.36″N | 098°51′10.18″E | 1324 | 500 plants, 300 inflorescences |
| Shan-pa (SP) | 26°57′16.73″N | 098°51′59.30″E | 1208 | 1000 plants, 300 inflorescences |
| Jia-ke-ding (JKD) | 26°46′14.85″N | 098°53′18.10″E | 1154 | 800 plants, 400 inflorescences |
| Pi-he(PH) | 26°40′52.55″N | 098°53′50.05″E | 1132 | 300 plants, 100 inflorescences |

**Table S2 Aphids on plants with budding inflorescence during the first survey (March 7-17, 2012)**

| Populations | | Aphids | |
| --- | --- | --- | --- |
| Name | The number of plants | The number of aphids | The percent of infection |
| Songta (ST) | 100 | 0 | 0 |
| Shuanla(SL) | 50 | 0 | 0 |
| Bendanqiao (BDQ) | 22 | 56 | 36.3% |
| Bendan (BD) | 25 | 1 | 4% |
| Pu-la-ding (PLD) | 100 | 234 | 14% |
| Ma-ji (MJ) | 100 | 31 | 16% |
| Shi-yue-liang (SYL) | 100 | 36 | 11% |
| Shan-pa (SP) | 100 | 1 | 1% |
| Jia-Ke-ding (JKD) | 100 | 68 | 8% |
| Pi-he(PH) | 100 | 237 | 11% |

**Table S3 Aphids on plants with blooming inflorescence during the first survey (April 11–13, 2012)**

| Population | | aphids | |
| --- | --- | --- | --- |
| name | The number surveyed plants | The number of aphids | The percent of infection |
| Songta (ST) | No blooming plants |  |  |
| Shuanla(SL) | No blooming plants |  |  |
| Bendanqiao (BDQ) | No blooming plants |  |  |
| Bendan (BD) | No blooming plants |  |  |
| Pu-la-ding (PLD) | 71 | 504 | 30.99% |
| Ma-ji (MJ) | No blooming plants |  |  |
| Shi-yue-liang (SYL) | 62 | 51 | 3.23% |
| Shan-pa (SP) | 100 | 0 | 0 |
| Jia-Ke-ding (JKD) | 100 | 67 | 4% |
| Pi-he (PH) | 77 | 9 | 5.19% |

**Table S4 Aphids on plants with blooming inflorescence during the second survey**

| Population | | aphids | |
| --- | --- | --- | --- |
| name | The number surveyed plants | The number of aphids | The percent of infection |
| Songta (ST) | 50 | 2 | 4% |
| Shuanla(SL) | 50 | 2 | 4% |
| Bendanqiao (BDQ) | 32 | 4 | 6.7% |
| Bendan (BD) | 100 | 18 | 11% |
| Pu-la-ding (PLD) | 100 | 48 | 12% |
| Ma-ji (MJ) | 100 | 33 | 19% |
| Shi-yue-liang (SYL) | * | * | * |
| Shan-pa (SP) | 100 | 3 | 2% |
| Jia-Ke-ding (JKD) | 100 | 18 | 7% |
| Pi-he(PH) | No blooming plants |  |  |

*This population was destroyed due to road construction

**Table S5. Taxa, voucher and GenBank accession numbers of Epipactis used in this study**

| Species | Voucher | *rbcL* | *matK* | ITS |
| --- | --- | --- | --- | --- |
| *E. albensis* | - | FJ454878 | - | AY154384 |
| *E. atrorubens* | - | JX088503 | JN894244 | JN847403 |
| *E. duriensis* | -- | - | - | AY351377 |
| *E. fageticola* | - | FJ454877 | - | AY351382 |
| *E. flava* | - | - | - | FJ454869 |
| *E. helleborine* | Jin Xiaohua, Jin Weitao, Xu Songzhi 13207 (PE) | KF419090* | KF419095* | KF419100* |
| *E. leptochila* | - | FJ454879 | JN894967 | FJ454870 |
| *E. lustitanica* | - | - | - | AY351381 |
| *E. mairei* | Jin Xiaohua, Jin Weitao, Xu Songzhi 13128 (PE) | KF419091* | KF419096* | KF419101* |
| *E. microphylla* | - | JX094818 | - | FR750399 |
| *E. muelleri* | - | FJ454881 | - | FJ454871 |
| *E. papillosa* | Jin Xiaohua, Jin Weitao, Xu Songzhi 13284 (PE) | KF419092* | KF419097* | KF419102* |
| *E. palustris* | - | FJ454882 | JN896120 | AY146448 |
| *E. phyllathes* | - | JN891262 | JN894394 | - |
| *E. pseudopurpurea* | - | - | - | JN847412 |
| *E. purpurata* | - | JN094816 | - | JN847416 |
| *E. roylenana* | Jin Xiaohua 20120623 (PE) | KF419093* | KF419098* | KF419103* |
| *E. thunbergii* | - | JF972911 | JX903645 | - |
| *E. voethii* | - |  | - | FR750400 |
| *E. veratrifolia* (China) | Jin Xiaohua 13048 (PE) | KF419094* | KF419099* | KF419104* |
| *E. veratrifolia* (Pakistan) | FPH-0655 (PE) | KF724436 | KF727437 | KF727435 |
| *E. veratrifolia* | - | JN005445 | JN004425 | JN114497 |
| **Outgroups** |  |  |  |  |
| *Aphyllorchis sp.* | - | - | JN706691 | FJ454867 |
| *Cephalanthera damasonii* | - | AF074123 | AY368396 | AY146446 |
| *C. longifolia* | - | JX051381 | - | AY369083 |
| *C. rubra* | - | JX088502 | - | AY369084 |
| *Limodorum abortivum* | - | JX051378 | - | AY351378 |
| *Listera ovata* | - | JX051379 | JN896261 | FJ694841 |
| *Neottia nidus-avis* | - | - | EF079303 | AY351383 |

Note: * represents the data obtained in this study.

**Table S6 Primers used for amplification in this study**

| Loci | Name | Sequence (5’-3’) |
| --- | --- | --- |
| rbcL | 1F | ATG TCA CCA CAA ACA GAA AC |
|  | 1360R | CTT CAC AAG CAG CAG CTA GTT C |
| matK | 19F | CGT TCT GAC CAT ATT GCA CTA TG |
|  | 390F | CGA TCT ATT CAT TCA ATA TTT C |
|  | trnK-2R | AAC TAG TCG GAT GGA GTA G |
| ITS | 17SE | ACG AAT TCA TGG TCC GGT GAA GTG TTC G |
|  | 26SE | TAG AAT TCC CCG GTT CGC TCG CCG TTA C |

**Table S7. Pollination systems of *Epipactis* and *Cephalanthera***

| Species | Pollinators | Literature |
| --- | --- | --- |
| *Cephalanhera longifolia* | Solitary bees | [[15](#_ENREF_15)] |
| *C. rubra* | Solitary bees | [[16](#_ENREF_16)] |
| *Epipactis atrorubens* | Bumble-bees | [[5](#_ENREF_5)] |
| *E. helleborine* | Social wasp (parasitic) | [[6](#_ENREF_6)] |
| *E. microphylla* | Autogamy | [[7](#_ENREF_7)] |
| *E. muellerie* | Autogamy | [[17](#_ENREF_17)] |
| *E. palustris* | Facultative autogamy, ants, fly, bee | [[5](#_ENREF_5), [8](#_ENREF_8), [9](#_ENREF_9)] |
| *E. purpurata* | Social wasp (parasitic) | [[5](#_ENREF_5), [13](#_ENREF_13)] |
| *E. royleana* | Hoverflies | ^#^ [[10](#_ENREF_10)] |
| *E. thunbergii* | Hoverflies | [[14](#_ENREF_14)] |
| *E. veratrifolia* | Hoverflies | [[11](#_ENREF_11), [12](#_ENREF_12)] |

^#^*Epipactis gigantea* and *E. royleana* are considered as identical in floral morphology (sensu Rose et al 1999 [[18](#_ENREF_18)]).

**Table S8 Statistics from the analyses of the various datasets**

| Data set | Taxon | Total length | Parsimony-informative characters | Model | Tree length | CI | RI |
| --- | --- | --- | --- | --- | --- | --- | --- |
| *rbcL* | 24 | 1290 | 20 | HKY | 125 | 0.960 | 0.839 |
| *matK* | 15 | 1429 | 34 | TVM+I | 132 | 0.917 | 0.838 |
| ITS | 27 | 631 | 121 | SYM+G | 297 | 0.781 | 0.848 |
| Three loci | 29 | 3350 | 175 | GTR+I+G | 559 | 0.848 | 0.845 |

**Figure S1** Habitat and floral organs of *Epipactis veratrifolia*. a) Habitat of *E. veratrifolia* along the Salween bank; b) hypochile, epichile, column and anther cap of *E. veratrifolia,* arrow indicating anther cap；c) Larva on dorsal sepal, aphid on lateral sepal (arrows indicate aphid and larva); d) Aphids and egg on flowers (arrows indicate aphids and egg. For sense of scale, a, the plant in bloom averages 40-60 cm in height; b, the length of anther cap averages 3 mm; c, the dorsal sepal averages 12 mm; d, egg length averages 0.7 mm.

**Figure S2** Distribution of *E. veratrifolia* in Eastern Himalayas along Salween.

Literature

1. Thompson JD, Gibson TJ, Plewniak F, Jeanmougin F, Higgins DG: **The CLUSTALX windows interface: flexible strategies for multiple sequence alignment aided by quality analysis tools**. *Nucleic Acids Research* 1997, **25**(24):4876-4882.

2. Hall TA: **BioEdit: a user-friendly biological sequence alignment editor and analysis program for Windows 95/98/NT**. *Nucleus Acids Symposium Series* 1999, **41**:95-98.

3. Swofford DL: **PAUP^*^: Phylogenetic analysis using parsimony (*and other methods), version 4.0b10.** Sunderland, Massachusetts: Sinauer; 2003.

4. Ronquist F, Huelsenbeck, J.P.: **MrBayes 3: Bayesian phylogenetic inference under mixed models.** *Bioinformatics* 2003, **19**:1572-1574.

5. Jakubska-Busse A, Kadej M: **The pollination of *Epipactis* Zinn,1757 (Orchidaceae) species in entral Europe -the significance of chemical attractants, floral morphology and conconitant insects**. *Acta Societatis Botanicorum Poloniae* 2011, **80**(1):49-57.

6. Brodmann J, Twele R, Francke W, Hoelzler G, Zhang Q-H, Ayasse M: **Orchids mimic green-leaf volatiles to attract prey-hunting wasps for pollination**. *Current Biology* 2008, **18**(10):740-744.

7. Bonatti PM, Sgarbi E, Del Prete C: **Gynostemium micromorphology and pollination in *Epipactis microphylla* (Orchidaceae)**. *Journal of Plant Research* 2006, **119**(5):431-437.

8. Talalaj I, Brzosko E: **Selfing potential in *Epipactis palustris*, *E. helleborine* and *E. atrorubens* (Orchidaceae)**. *Plant Systematics and Evolution* 2008, **276**(1-2):21-29.

9. Brantjes NMB: **Ant, bee and fly pollination in *Epipactis palustris* (L.) Crantz (Orchidaceae)**. *Acta Botanica Neerlandica* 1981, **30**(1-2):59-68.

10. van der Cingel NA: **An atlas of orchid pollination: America, Afirca, Asia and Australia**. Rotterdam, Netherlands: A.A. Balkema; 2001.

11. Ivri Y, Amotes. D: **Pollination ecology of *Epipactis consimilis* Don. (Orchidaceae) in Israel**. *New Phytologist* 1977, **79**(1):173-177.

12. Stökl J, Brodmann J, Dafni A, Ayasse M, Hansson BS: **Smells like aphids: orchid flowers mimic aphid alarm pheromones to attract hoverflies for pollination**. *Proceedings of the Royal Society B-Biological Sciences* 2011, **278**(1709):1216-1222.

13. Ehlers BK, Olesen JM: **The fruit-wasp route to toxic nectar in *Epipactis* orchids**. *Flora* 1997, **192**:223-229.

14. Sugiura N: **Pollination of the orchid *Epipactis thunbergii* by syrphid flies (Diptera: Syrphidae)**. *Ecological Research* 1996, **11**:249-255.

15. Amotes D, Yariv I: **The flower biology of *Cephalanthera longifolia* (Orhidaceae)-Pollen Imitation and Facultative Floral Mimicry**. *Plant systematics and Evolution* 1981, **137**:229-240.

16. Nilsson LA: **Mimesis of bellflower (*Campanula*) by the red helleborine orchid *Cephalanthera rubra***. *Nature* 1983, **305**(27):799-800.

17. Squirrell J, Hollingsworth PM, Bateman RM, Tebbitt MC, Hollingsworth ML: **Taxonomic complexity and breeding system transitions: conservation genetics of the *Epipactis leptochila* complex (Orchidaceae)**. *Molecular Ecology* 2002, **11**(10):1957-1964.

18. Rose TK, Bhattacharjee SK, Basak PDUC: **Orchids of India**. Calcutta, India: Nata Prokash; 1999.
