# Supplementary figures and images for: The evolution of floral deception in Epipactis veratrifolia (Orchidaceae): from indirect defense to pollination
Source: BMC Plant Biol. 2014 Mar 12;14:63. doi: 10.1186/1471-2229-14-63 (PMC4007573; doi:10.1186/1471-2229-14-63)

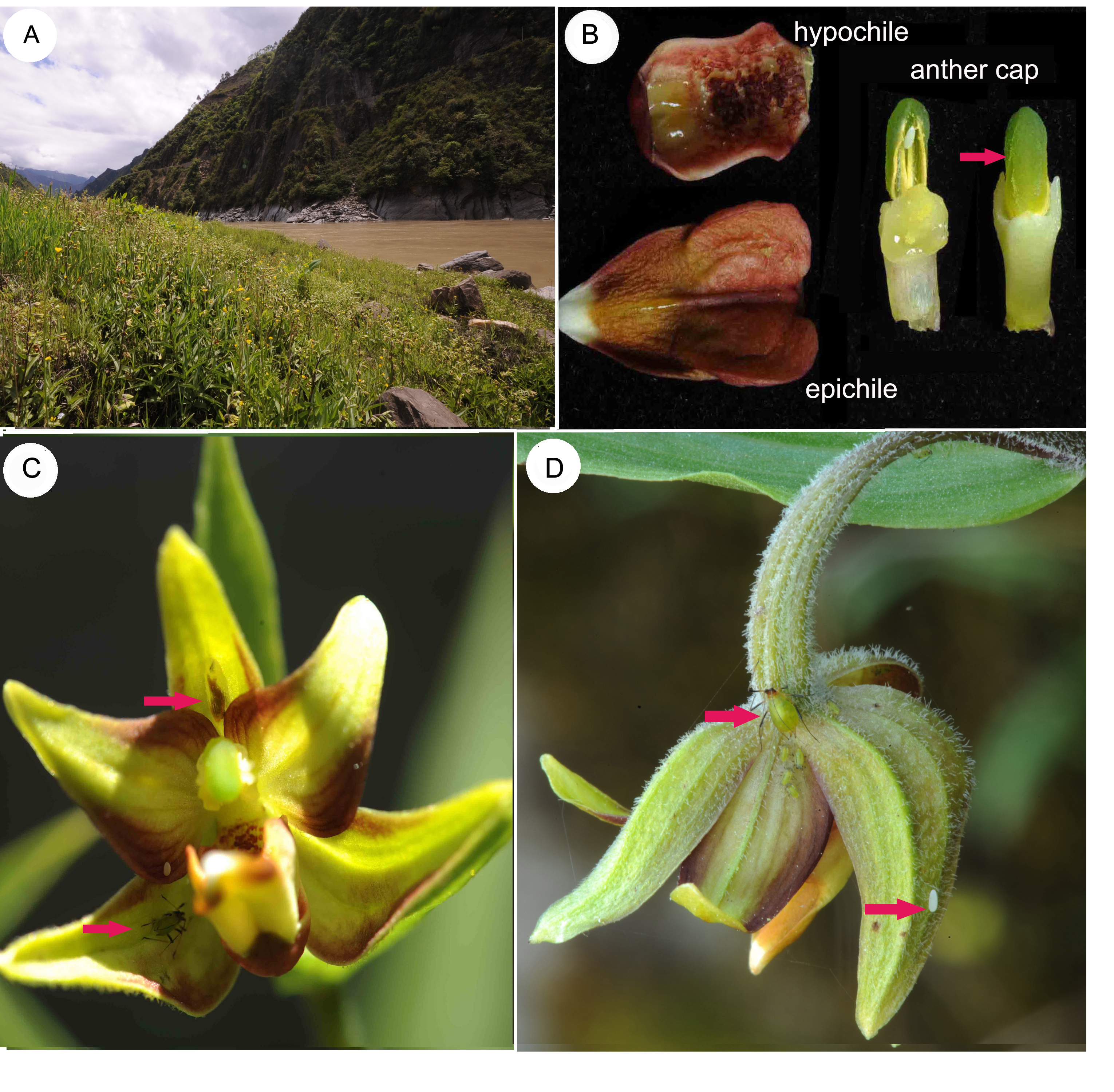

Supplement: Additional file 2: Figure S1 — Habitat and floral organs of Epipactis veratrifolia. A) Habitat of E. veratrifolia along the Salween bank; B) hypochile, epichile, column and anther cap of E. veratrifolia, arrow indicating anther cap; C) Larva on dorsal sepal, aphid on lateral sepal (arrows indicate aphid and larva); D) Aphids and egg on flowers (arrows indicate aphids and egg. For sense of scale, A, the plant in bloom averages 40-60 cm in height; B, the length of anther cap averages 3 mm; C, the dorsal sepal averages 12 mm; D, egg length averages 0.7 mm. [file 1471-2229-14-63-S2.tiff]

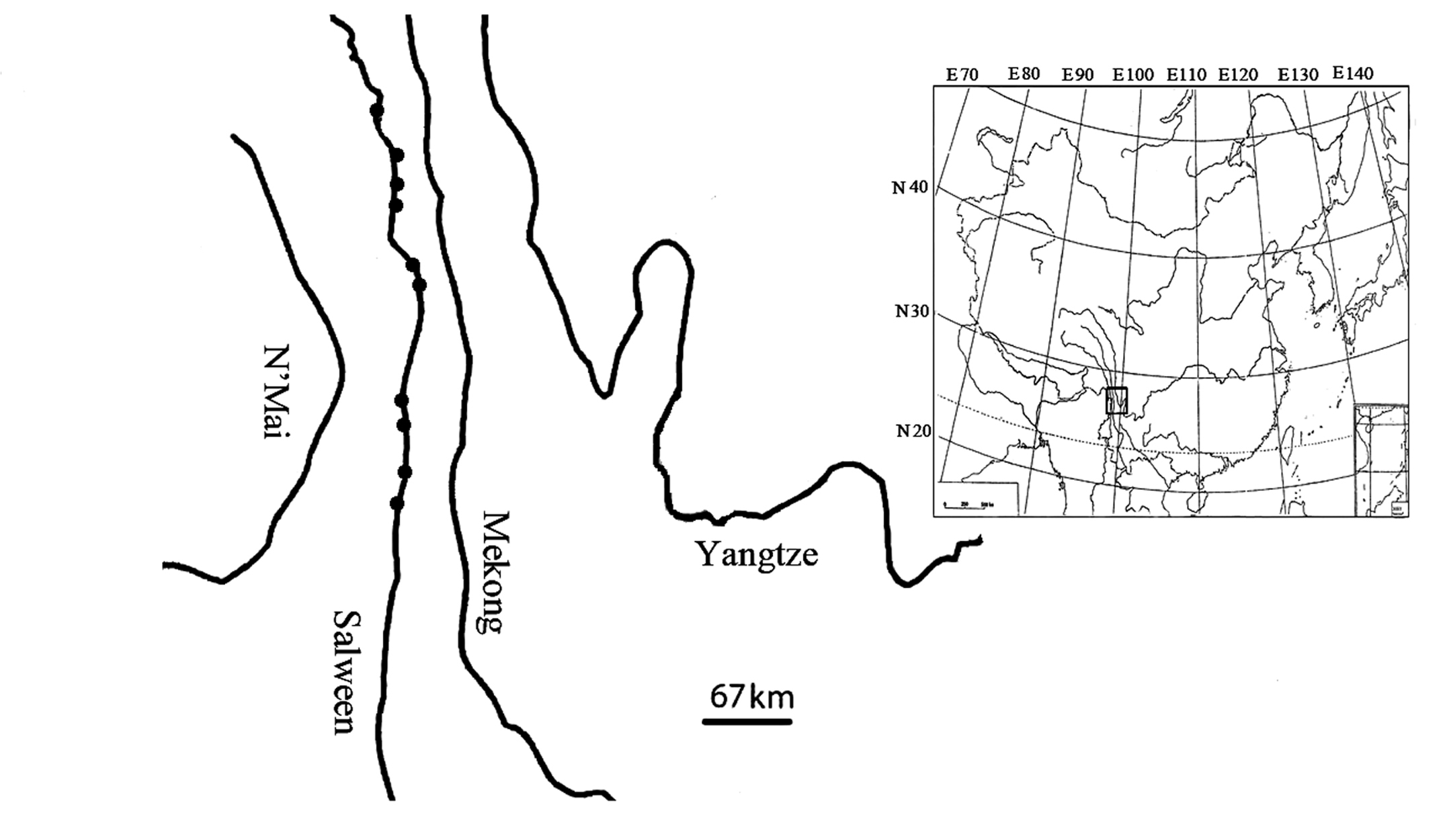

Supplement: Additional file 3: Figure S2 — Distribution of E. veratrifolia in Eastern Himalayas along Salween. [file 1471-2229-14-63-S3.jpeg]
